# A multiplex loop-mediated isothermal amplification assay for rapid screening of *Acinetobacter baumannii* and D carbapenemase *OXA-23* gene

**DOI:** 10.1042/BSR20180425

**Published:** 2018-09-07

**Authors:** Rungong Yang, Honghong Zhang, Xiaoxia Li, Ling Ye, Meiliang Gong, Jinghui Yang, Jihong Yu, Jie Bai

**Affiliations:** 1 Department of Orthopedics, First Affiliated Hospital, Chinese PLA General Hospital, Beijing 100048, China; 2 Institute of Geriatrics/Key Laboratory of Normal Aging and Geriatrics, Chinese PLA General Hospital, Beijing 100853, China; 3 Clinical Lab of Nanlou Department, Chinese PLA General Hospital, National Clinical Research Center for Geriatric Diseases, Beijing 100853, China

**Keywords:** Acinetobacter baumannii, D carbapenemase OXA-23 gene, multiplex loop-mediated isothermal amplification, screen

## Abstract

**Background**: *Acinetobacter baumannii* is a health burden responsible for various nosocomial infections, and bacteremia in particular. The resistance of *A. baumannii* to most antibiotics including carbapenem has increased. OXA-23-producing *A. baumannii* is the chief source of nosocomial outbreaks with carbapenem-resistant *A. baumannii*. Successful antibiotic treatment relies on the accurate and rapid identification of infectious agents and drug resistance. Here, we describe a multiplex loop-mediated isothermal amplification (LAMP) assay for simultaneous and homogeneous identification for *A. baumannii* infection screening and drug-resistance gene detection. **Methods**: Four primer pairs were designed to amplify fragments of the *recA* gene of *A. baumannii* and the *oxa-23* gene. The reaction with a 25 μl of final volume was performed at 63°C for 60 min. For comparative purposes, we used a traditional method of bacterial identification to evaluate assay efficacy. **Results**: The multiplex LAMP assay enables simultaneous and homogeneous detection of the *recA* gene of *A. baumannii* and the *oxa-23* gene and requires less than 21 min with no pre-requisite for DNA purification prior to the amplification reaction. The detection is specific to *A. baumannii*, and the coincidence rate of the multiplex LAMP and the traditional method was 100%. **Conclusions**: Our data indicate that the multiplex LAMP assay is a rapid, sensitive, simultaneous and homogeneous method for screening of *A. baumannii* and its drug-resistance gene.

## Introduction

*Acinetobacter baumannii*, an opportunistic pathogen, is a health burden responsible for various nosocomial infections, and bacteremia in particular [1–3]. Due to the wide application of broad-spectrum antibiotics, resistance of *A. baumannii* to most antibiotics has increased, and the emergence of carbapenem-resistant (CR) *A. baumannii* has been described as a sentinel event of clinically relevant antimicrobial resistance [4]. Recent research shows that bacteremia caused by CR *A. baumannii* typically leads to increased mortality and prolonged hospital stays [5–7].

Among CR *A. baumannii* isolates, production of acquired carbapenem-hydrolyzing class D β-lactamases (CHDLs), including those belonging to the OXA-23, -24 and -58 families, and overproduction of the intrinsic class D β-lactamase OXA-51 family are the most prevalent mechanisms for carbapenem resistance [8]. OXA-23-producing *A. baumannii* is the chief source of nosocomial outbreaks with CR *A. baumannii* [9,10].

The propensity of *A. baumannii* to be multidrug-resistant or extensively drug-resistant presents therapeutic and infection control challenges. Successful antibiotic treatment of infections relies on accurate and rapid identification of the infectious agents and drug resistance. Various culture-based methods and susceptibility testing are time-consuming and delay confirmation of the strain and the start of appropriate antibiotic therapy. Some recently developed molecular techniques (loop-mediated isothermal amplification (LAMP)) are rapid and relatively simple to use and have been described for identifying DNA directly from clinical specimens [11,12].

We describe the development of a multiplex LAMP assay for simultaneous and homogeneous identification for *A. baumannii* infection screening and drug-resistance gene detection. To the best of our knowledge, this is the first study that outlines this approach. The present study was performed at the Chinese PLA General Hospital from 2016 to 2017, and data show that the multiplex LAMP assay is a rapid, sensitive and direct method for screening of *A. baumannii* and its drug-resistance gene and requires less than 21 min with no pre-requisite for DNA purification prior to the amplification reaction.

## Materials and methods

### Definitions

CR *A. baumannii* is defined if an *A. baumannii* isolate was resistant to both imipenem and meropenem [13].

### Bacterial isolates

All consecutive and nonduplicate clinical isolates from hospitalized patient samples (such as gastric or drainage fluid, urine, blood and sputum) were collected including *Candida albicans* isolates, *Candida tropicalis* isolates, *Candida parapsilosis* isolates, *Klebsiella pneumonia* isolates, *Enterobacter cloacae* isolates, *Escherichia coli* isolates, *Streptococcus viridans* isolates, *Micrococcus* isolates, *Enterococcus* isolates, coagulase negative *Staphylococcus* isolates, *A. baumannii* isolates, *Stenotrophomonas maltophilia* isolates and *Pseudomonas aeruginosa* isolates. Standard microbiologic methods were used to isolate and identify bacteria from clinical specimens. Bacterial antimicrobial susceptibility testing was performed in accordance with the methodology of the Clinical and Laboratory Standards Institute 2014 [14].

CR *A. baumannii* was identified using VITEK 2 Compact (Bio Mérieux SA, Marcy l‘Etoile, France).

### Primer design and LAMP method

The primer pairs (outer and inner) used for identification of *A. baumannii* and its antimicrobial susceptibility were designed to amplify fragments of the *recA* gene of *A. baumannii* and the *oxa-23* gene using LAMP Designer software (http://www.optigene.co.uk) (OptiGene Ltd, United Kingdom) (Table 1).

**Table 1:** Primer sets used for specific isothermal amplification of *A. baumannii*

Primer	Sequence	Amplification temperature (°C)	Product size of endonuclease digestion (bp)
oxa-23-F3	AAAGACATGACACTAGGAGAA	63	136
oxa-23-B3	GCGTAACCTTTAATGGTCCT		
oxa-23-FIP	ATCAAGACCGATACGTCGC***GAATTC***GCCATGAAGCTTTCTGCA		
oxa-23-BIP	AGAAGTAAAACGTATTGGTTTCGGT***GAATTC***CAACCAGAAATTATCAACCTG		
recA-F3	CCTTCATTGATGCTGAGCA	63	210
recA-B3	AAGACGCGCTTGTAGACC		
recA-FIP	GCTCACCATTGTCGGGTTGT***GAATTC***ACGCAAACTTGGTGTAG		
recA-BIP	CGCAATTGATTTAATCGTTGTGGAC***GAATTC***ATATGAGAGTCACCCATCTC		

*Bold italics are sites for restriction endonuclease *Eco*RI.

Bacterial genomic DNA was collected by boiling. Single colonies were collected in 500 μl of sterile purified water and then serially diluted. Diluted bacterial solutions were boiled for 30 min and centrifuged at 10,000×***g*** for 8 min. Supernatants (total genomic DNA) were aliquoted for storage at −80°C for later study.

LAMP was performed using the commercially available Loopamp DNA Amplification Kit (Isothermal Master Mixes and Bst DNA Polymerases, OptiGene Ltd, United Kingdom), and Loopamp DNA Amplification Kit containing Bst DNA Polymerases (LAMP) (Eiken China Co. Ltd, Shanghai, China). Three different instruments that allow isothermal amplification of DNA and RNA, Genie II (OptiGene Ltd, United Kingdom) and ROCHE LightCycler 480 (Roche Diagnostics Ltd., Rotkreuz, Switzerland) with target detection using a fluorescent measurement, and Loopamp Realtime Turbidimeter LA-320c (Eiken Chemical Co. Ltd, Tokyo, Japan) with target detection using a turbidimetric assay, were used according to the manufacturer’s instructions.

The reaction mixture was prepared by mixing 1 μl each of 5 μmol l^−1^ outer primers F3 and B3, 1 μl each of 40 μmol l^−1^ inner primers FIP and BIP, 15 μl of mixed buffer and 3.5 μl of double-distilled H_2_O. The reaction with a 25 μl of final volume was initiated by adding 2.5 μl of template and incubated at 63°C for 60 min. When the *recA* and *oxa-23* genes of *A. baumannii* were simultaneously and homogeneously amplified in a single reaction tube, primer pairs for fragment amplification were premixed (ratio 1.3:1) and added to the reaction tube as described. Amplification of the positive DNA yielded a sigmoidal amplification curve, and the negative control tube had no measurable fluorescence or turbidity as indicated by a flat line in the plot. The desired products were confirmed by agarose gel electrophoresis. A negative control was performed using sterile water instead of DNA template.

### *Baumannii* and carbapenem-resistance

To evaluate specificity of fragment amplification of the *recA* gene for confirming *A. baumannii*, a total of 22 bacterial strains were used including two *C. albicans* isolates, a single *C. tropicalis* isolate, a single *C. parapsilosis* isolate, two *K. pneumonia* isolates, a single *E. cloacae* isolate, a single *E. coli* isolate, two *S. viridans* isolates, a single *Micrococcus* isolate, a single *Enterococcus* isolate, a single coagulase negative *Staphylococcus* isolate, three *A. baumannii* isolates, a single *P. mirabilis* isolate, two *S. maltophilia* isolates and three *P. aeruginosa* isolates. Bacterial colonies were boiled for 30 min to obtain bacterial genomic DNA. Amplifications were carried out using a Loopamp DNA Amplification Kit on Loopamp Realtime Turbidimeter LA-320c. The specificity of the LAMP assay for *A. baumannii* was evaluated by comparing results identified by standard methods. The detection limit was ascertained using serial dilutions of bacteria. These experiments were replicated to ensure reproducibility.

### Multiplex LAMP assay

Two *A. baumannii* isolates with positive *oxa-23* gene amplification were used to perform the multiplex LAMP assay that simultaneously and homogeneously amplified *recA* and *oxa-23* genes in a single reaction tube with four pairs of primers. The multiplex LAMP assay was carried out using the commercially available Loopamp DNA Amplification Kit (Isothermal Master Mixes and DNA Polymerases) on a Genie II or a ROCHE LightCycler 480.

## Results

**Figure 1:**
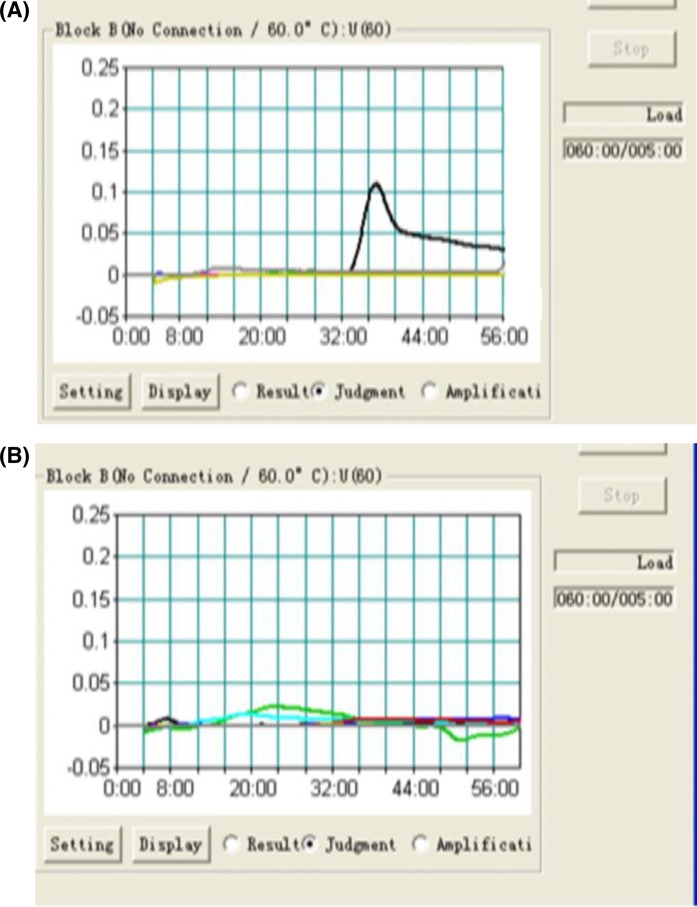
LAMP assay for *A. baumannii* amplification of the *recA* gene using a Loopamp Realtime Turbidimeter LA-320c. (**A**) *A. baumannii*, amplification curve observed within 36 min of amplification. (**B**) *P. aeruginosa, S. maltophilia, S. aureus* and *P. mirabilis*, no amplification curve observed with *recA* gene amplification.

**Figure 2:**
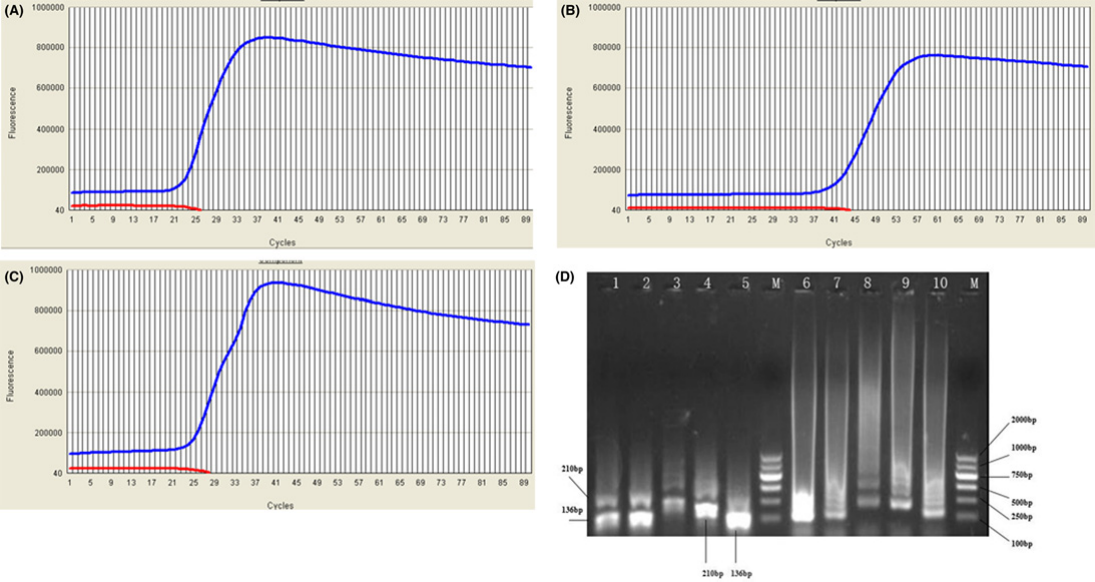
Simultaneous homogeneous amplification of *recA* and *oxa-23* gene in a single reaction tube using ROCHE LightCycler 480 (**A**) Amplification curve of the *recA* gene; (**B**) amplification curve of the *oxa-23* gene; (**C**) specific multiplex amplification curve of the *recA* and *oxa-23* genes; (**D**) agarose gel electrophoresis of amplified *A. baumannii*-specific products and restriction fragments with *Eco*RI digestion. Amplified *oxa-23* gene fragment is 136 bp and the *recA* gene fragment is 210 bp. Lane M: DNA molecular weight marker; Lanes 1 and 2: restriction fragments of mixed products of multiplex amplification with *Eco*RI digestion; Lanes 3 and 4: restriction fragments of amplifying the *recA* gene with *Eco*RI digestion; Lane 5: restriction fragments of amplifying the *oxa-23* gene with *Eco*RI digestion; Lanes 6 and 7: mixed products of the multiplex amplification of *recA* and *oxa-23* genes; Lanes 8 and 9: amplified products of the *recA* gene; Lane 10: amplified products of the *oxa-23* gene.

**Figure 3:**
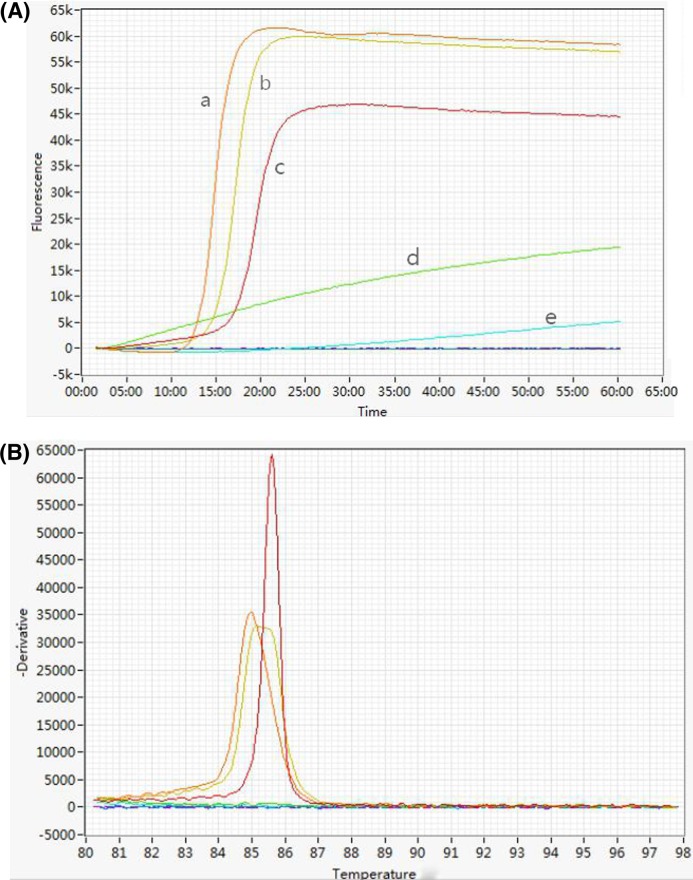
Amplification and anneal curves of amplified products of *recA* and *oxa-23* genes of *A. baumannii* using a Genie II (**A**) Amplification curves; (**B**) corresponding annealing curves of amplified products. (a) amplification curve of the *oxa-23* gene; (b) multiplex amplification curve with a primer ratio of 1:1 of the *recA* to *oxa-23* gene; (c) amplification curve of the *recA* gene; (d) and (e) negative controls for *recA* and *oxa-23* gene amplification, respectively.

**Figure 4:**
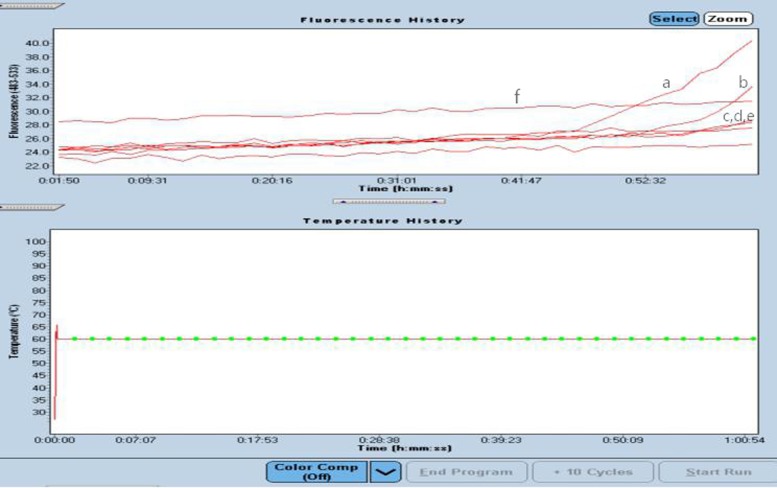
Amplification curves of the *recA* gene of serially diluted *A. baumannii* using a ROCHE LightCycler 480. (a) 3000 copies/μl; (b) 300 copies/μl; (c) 30 copies/μl; (d) 3 copies/μl; (e) 0.3 copies/μl; (f) 0.03 copies/μl

We developed a specific multiplex LAMP assay for measuring *A. baumannii* and carbapenem-resistance. This assay facilitated amplification of the *recA* gene of *A. baumannii* within 36 min of initiation using the Loopamp Realtime Turbidimeter LA-320c (Figure 1), 25 min using a ROCHE LightCycler 480 (Figure 2A) and 20 min with a Genie II (Figure 3A). Using serial dilutions, ∼300 copies μl^−1^ of specific nucleic acid targets were assayed after 25 min using the ROCHE LightCycler 480 (Figure 4). Of the 22 identified bacterial isolates, three were positive for *recA* gene amplification and identified as *A. baumannii* using classical culture-based methods. No amplification curve was observed with the 19 non-*Acinetobacter baumannii* bacterial DNA samples. An analysis of specificity associated with the LAMP assay confirmed that only target *A. baumannii* DNA generated a positive result. DNA extracted from *C. albicans, C. tropicalis, C. parapsilosis, K. pneumoniae, E. cloacae, E. coli, S. viridans, Micrococcus, Enterococcus*, coagulase negative *Staphylococcus, P. aeruginosa*, mucoid *P. aeruginosa, S. aureus, Cryptococcus* and *S. maltophilia* were negative with the LAMP assay. This confirmed specificity for *A. baumannii* using the LAMP assay amplifying the *recA* gene, and that *A. baumannii* could be identified by amplifying the *recA* gene.

All three isolates with positive *recA* gene amplification were resistant to imipenem and meropenem according to standard antibiotic susceptibility tests and two were *oxa-23* gene-positive (Figure 2B). When the multiplex amplification was conducted on the Genie II, a positive curve appeared within 18 min (Figure 3A). The multiplex amplification products were confirmed by agarose gel electrophoresis and two DNA bands, a 136-bp fragment for the *oxa-23* gene and a 210-bp fragment for the *recA* gene appeared. Conversely, only one gene was amplified, yielding an amplification product (Figure 2D).

**Figure 5:**
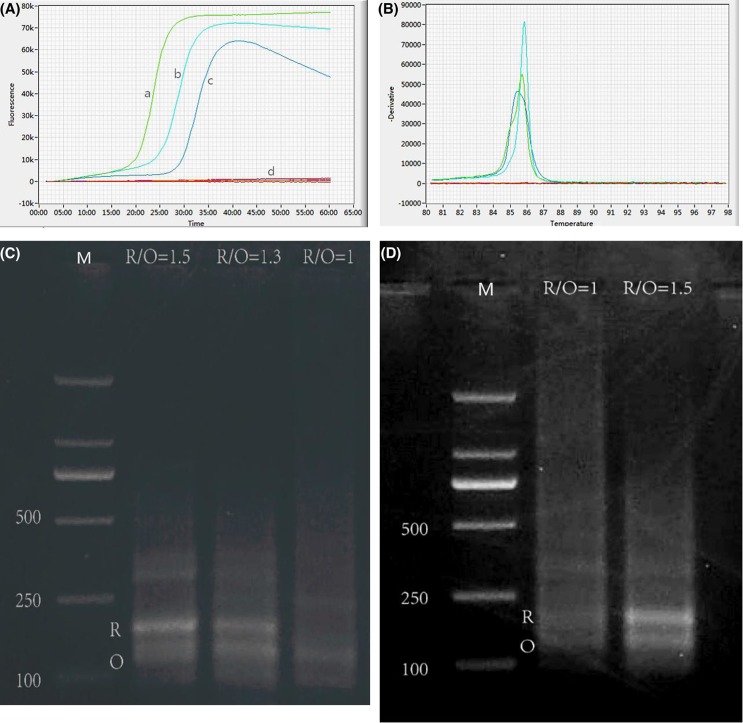
Multiplex amplification curves with different primer ratios of *recA* to *oxa-23* genes and corresponding annealing curves of amplified products using a Genie II (**A**) Multiplex amplification curves of *recA* and *oxa-23* genes with different primer ratios; (**B**) corresponding annealing curves of multiplex amplification products; (**C** and **D**) agarose gel electrophoresis of restriction fragments of multiplex amplification products with *Eco*RI digestion; (a), (b) and (c) the primer ratio of the *recA* to *oxa-23* genes was 1.3:1, 1.5:1 and 1:1, respectively; (d) negative control for multiplex amplification with a primer ratio of 1.3:1 of the *recA* to the *oxa-23* gene; R/O: primer ratio of the *recA* to *oxa-23*; R: restriction fragment of the *recA* gene amplified; O: restriction fragment of the *oxa-23* gene amplified; M: DNA molecular weight marker.

**Figure 6:**
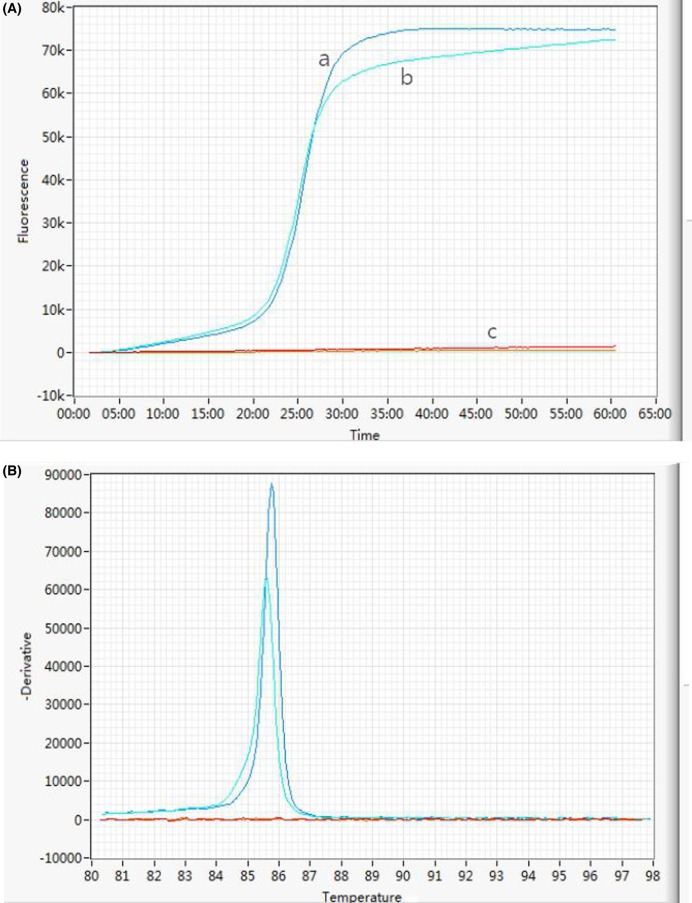
Simultaneous and homogeneous amplificaion of the *recA* and *oxa-23* gene in a single reaction tube using Genie II to identify *A. baumannii* and antimicrobial resistance (**A**) Amplification curves; (**B**) corresponding annealing curves of amplified products; (a) and (b) primer ratio of *recA* to *oxa-23* gene was 1.5:1 and 1:1, respectively; (c) negative control.

We observed that the annealing temperature of *recA* gene products was ∼0.7°C higher than the *oxa-23* gene (Figure 3B), and a greater ratio of recA to oxa-23 primer pairs, the greater the annealing temperature of mixed products of both genes in the multiplex amplification (Figures 5 and 6). The annealing temperature of mixed products was not specific (85.3–85.8°C) depending on the ratio of primer pairs for both genes. Additionally, when the multiplex amplification was carried out on a ROCHE LightCycler 480, a positive curve presented a specific superposition of two curves (Figure 2C). Data show that identification of *A. baumannii* and carbapenem resistance can be done simultaneously and homogeneously in a short time.

## Discussion

The *recA* gene is an important housekeeping gene in most bacteria. Its function mainly involves homologous recombination, DNA repair, the SOS response, combining single-stranded DNA, DNA unraveling and seeking homologous loci for homologous recombination [15–16]. The *recA* gene has a high discriminatory power for differentiating species and subspecies within various bacterial genera, such as *Vibrio* and *Lactobacillus* species [17–21]. In 2012, *recA* sequences were used to develop a real-time PCR assay for discrimination of *S. pseudopneumoniae* from closely related species with excellent specificity [22]. A multiplex PCR targeting the 16S-23S rRNA intergenic spacer gene sequence and the *recA* gene sequence were also established to identify *A. baumannii* rapidly, which was effective for identifying reference strains and clinical isolates of *A. baumannii* in environmental specimens [23]. Thus, the *recA* gene is a confirmatory alternative tool for molecular identification and epidemiological studies.

Carbapenemases produced by *A baumannii* are mostly class D oxacillinases, although metalloenzymes of the IMP, VIM and NDM families have been detected in some well-defined geographical regions [8, 24–25]. Carbapenemase activity is also an intrinsic property of many class D β-lactamases, which are referred to as CHDLs. The first and most common subgroup of CHDLs is made of *OXA-23, OXA-27* and *OXA-49* [26], and the *blaOXA-23* gene is considered to be a significant cause of carbapenem resistance in *A. baumannii* worldwide [27–28].

Real-time PCR is sensitive and accurate for identifying carbapenemase genes [29–30]. LAMP could identify bacteria directly from clinical specimens (plasma) within 20 min without DNA purification [31]. Compared with PCR, the biggest advantage of LAMP is that nucleic acid amplification is carried out isothermally at a lower temperature and in clinical samples such as serum, swabs and whole blood with little or no sample preparation [31–32]. Some multiplex LAMP methods that involve homogeneous and heterogeneous amplification have been reported [33–36].

Here, we describe a multiplex LAMP assay to directly amplify *recA* and *OXA-23* genes simultaneously and homogeneously in real-time at 63°C, which enables detection within 18 min without DNA purification before the multiplex amplification reaction. Figure 4 shows that the detection limit of the LAMP assay for *A. baumannii* was 10^2^ CFU μl^−1^ for the strain without loop primers for the LAMP reaction. All *A. baumannii* strains were amplified, and 19 non-*baumannii Acinetobacter* strains produced negative reactions. We also used the traditional method of bacterial identification to evaluate the newer assay, and the coincidence of both was 100%. These data indicated that the assay provides a sensitive, specific and simple diagnostic tool for *A. baumannii* infection*.*

Tone and co-workers investigated the influences of *T*_m_ values of LAMP products and found that inorganic pyrophosphatase (PPase) reduced the *T*_m_ value variances and allowed differentiation of pathogenic agents using an annealing curve analysis for the LAMP method [37]. However, annealing curve analysis is difficult to apply to the multiplex LAMP assay because products of different target genes were similar in structure and length, all of which were dumbbell-like structures. Therefore, the *T*_m_ (annealing temperature) values for each LAMP product do not differ significantly. We report that one peak appeared in the annealing curve of the mixed products of two different target genes, which indicates a common *T*_m_ value for the mixed products generated from different target genes. However, the common *T*_m_ value ranged over a narrow interval depending on the proportion of various products is not stable and specific (Figures 3B, 5B and 6B).

Our assay is robust enough to screen suspected *A. baumannii* infection and simultaneously detect the *OXA-23* carbapenemase gene, which reduces the time to the diagnosis of infection and improves medical treatment. This assay could also be performed on various real-time nucleic acid amplification apparatuses with target detection using fluorescence (Genie II is an isothermal amplification instrument, and ROCHE LightCycler 480 is a universal PCR device), suggesting a significant advantage for on-site application and efficient screening and testing of clinical samples. More work is required to confirm the sensitivity and specificity of this assay and how to indicate multiplex detection being introduced into the LAMP method.

## Abbreviations

CHDL: carbapenem-hydrolyzing class D β-lactamase
LAMP: loop-mediated isothermal amplification
